# The Functions of Auxilin and Rab11 in *Drosophila* Suggest That the Fundamental Role of Ligand Endocytosis in Notch Signaling Cells Is Not Recycling

**DOI:** 10.1371/journal.pone.0018259

**Published:** 2011-03-23

**Authors:** Susan M. L. Banks, Bomsoo Cho, Suk Ho Eun, Ji-Hoon Lee, Sarah L. Windler, Xuanhua Xie, David Bilder, Janice A. Fischer

**Affiliations:** 1 Section of Molecular Cell and Developmental Biology and Institute for Cell and Molecular Biology, The University of Texas at Austin, Austin, Texas, United States of America; 2 Department of Molecular and Cell Biology, University of California, Berkeley, California, United States of America; University of Dayton, United States of America

## Abstract

Notch signaling requires ligand internalization by the signal sending cells. Two endocytic proteins, epsin and auxilin, are essential for ligand internalization and signaling. Epsin promotes clathrin-coated vesicle formation, and auxilin uncoats clathrin from newly internalized vesicles. Two hypotheses have been advanced to explain the requirement for ligand endocytosis. One idea is that after ligand/receptor binding, ligand endocytosis leads to receptor activation by pulling on the receptor, which either exposes a cleavage site on the extracellular domain, or dissociates two receptor subunits. Alternatively, ligand internalization prior to receptor binding, followed by trafficking through an endosomal pathway and recycling to the plasma membrane may enable ligand activation. Activation could mean ligand modification or ligand transcytosis to a membrane environment conducive to signaling. A key piece of evidence supporting the recycling model is the requirement in signaling cells for *Rab11*, which encodes a GTPase critical for endosomal recycling. Here, we use *Drosophila Rab11* and *auxilin* mutants to test the ligand recycling hypothesis. First, we find that *Rab11* is dispensable for several Notch signaling events in the eye disc. Second, we find that *Drosophila* female germline cells, the one cell type known to signal without clathrin, also do not require auxilin to signal. Third, we find that much of the requirement for *auxilin* in Notch signaling was bypassed by overexpression of both clathrin heavy chain and epsin. Thus, the main role of auxilin in Notch signaling is not to produce uncoated ligand-containing vesicles, but to maintain the pool of free clathrin. Taken together, these results argue strongly that at least in some cell types, the primary function of Notch ligand endocytosis is not for ligand recycling.

## Introduction

Virtually all signaling pathways have an endosomal component [1]. Notch signaling, however, is remarkable in its absolute dependence on endocytosis [2]–[7]. The Notch receptor and its ligands (Delta and Serrate in *Drosophila*) are transmembrane proteins [8]. Although the roles of ligand and receptor internalization are unclear, endocytosis is essential to both Notch signaling and signal reception. Most counterintuitive is the requirement for ligand endocytosis into the signaling cells. Two classes of models have been proposed to explain why ligand needs to be internalized in order to signal [2]–[7]. The “pulling” model proposes that endocytosis of ligand bound to the Notch receptor exerts a mechanical force that activates the receptor either by exposing a proteolytic cleavage site on the receptor extracellular domain, or by dissociating the subunits of the receptor heterodimer. In contrast, ligand is endocytosed prior to receptor binding in the “recycling” model, and via an endosomal pathway, it is returned to the plasma membrane either in an activated form that can bind ligand, or to a new membrane environment favorable to receptor interaction.

Several results support the pulling model. First, when separated from its transmembrane domain and secreted, the extracellular domain of Delta blocks Notch activation [9]. Second, the extracellular domains of Notch and Delta are sometimes found together in endosomes inside signaling cells [10], [11]. Third, structural studies suggest that the ADAM protease site on the Notch extracellular domain, which must be cleaved to activate the receptor, is exposed by ligand binding [12]. Finally, there is evidence that ligand internalization into signaling cells depends on the presence of Notch in adjacent cells [13]. There is also evidence in favor of the recycling model. For example, in some epithelial cells, the GTPase Rab11, which is required for endosomal recycling [14], is needed in signaling cells for signaling and for Delta recycling [15]–[17]. In addition, the ligand intracellular domain, which is normally ubiquitinated by specific ubiquitin ligases that are necessary for signaling and ligand endocytosis [18]–[26], may be replaced by the internalization and recycling signals from the vertebrate LDL receptor [27]. Finally, Delta transcytosis has been observed, and it is thought to relocate ligand to a site on the plasma membrane near Notch in the adjacent cell [15]–[17], [28].

The pulling and recycling models are not necessarily mutually exclusive. It has been proposed that two ligand internalization events are required, the first to activate ligand through recycling, and the second to activate the receptor on an adjacent cell through pulling [28], [29].

Epsin and auxilin are two endocytic proteins required in signaling cells for ligand endocytosis and signaling [27], [30]–[36]. Epsin, which has been shown to be an essential component of the Notch pathway in *C. elegans* [36] and vertebrates [37], as well as in *Drosophila* [27], [30], has binding sites for the plasma membrane, ubiquitin, clathrin, and other proteins present in clathrin-coated vesicles [38]. Although the mechanism of epsin function in Notch signaling is not well understood, studies of epsin in other contexts suggest that epsin probably links ubiquitinated ligand with endocytic vesicles [38]. Another endocytic protein, auxilin, is also required in Notch signaling cells in all *Drosophila* tissues tested [31]–[34]. Auxilin brings the ATPase Hsc70 to clathrin cages, and stimulates Hsc70 to uncoat clathrin coated vesicles [39]. At first glance, it would appear that the requirement for auxilin supports the recycling model; uncoating of newly internalized clathrin-coated vesicles containing ligand is prerequisite for trafficking of ligand through an endosomal pathway for recycling. However, it is also possible that auxilin is required only to maintain the pool of free clathrin, and not for production of uncoated vesicles [33]. In addition, it was shown recently that to send Delta signals, *Drosophila* female germline cells require epsin-mediated endocytosis, but not clathrin [40]. Vertebrate epsin is known to function in both clathrin-dependent and clathrin-independent endocytosis [41]–[43]. However, this result suggests the possibility that epsin function in Notch signaling is generally clathrin-independent, and thus the function of auxilin in signaling cells might be other than its characterized role in clathrin dynamics.

Here, we performed genetic experiments in *Drosophila* to test the roles of *Rab11* and *auxilin* in Notch signaling, and ultimately to test the recycling model. First, we found that *Rab11* is not required for Notch signaling events in the eye disc that require both epsin and auxilin. Second, we found that female germline cells that do not require clathrin in order to signal also do not require auxilin. Finally, we found that overexpression of both clathrin heavy chain and epsin suppress nearly completely the lethality and severe eye morphology defects of *auxilin* mutants. Taken together, the results argue strongly that in many cell types, ligand recycling is not the primary function of epsin-dependent ligand endocytosis by Notch signaling cells.

## Results

### *Rab11* was dispensable for Notch signaling events in the eye disc

We wanted to determine whether or not ligand recycling is required for Notch signaling during eye development. If so, it would be expected that the two GTPases Rab5 and Rab11 would both be required in signaling cells. Rab5 mediates fusion of early endosomes with the sorting endosome, an event required for trafficking through any endosomal pathway, and Rab11 is required for subsequent routing of an endosome through the recycling pathway [14]. First, we asked about one characterized event early in eye development, called R-cell restriction [30]. Photoreceptors R2/R5 and R3/R4 in early ommatidial preclusters signal via Delta to other precluster cells, preventing them from becoming ectopic photoreceptors (R-cells). When this signaling event fails (for example in hypomorphic *lqf* or *aux* mutants), ommatidia have one or several extra photoreceptors [30], [32], [33], [44]. When dominant negative *shibire* (encodes *Drosophila* dynamin) or *Delta* genes are expressed specifically in R2/R5 and R3/R4 using a *rough* (*ro*) gene expression vector, ommatidia in adult eyes have extra R-cells due to failure of R-cell restriction [30]. Using the same *ro* expression vector, we generated transgenes expressing dominant negative forms of *Rab5* or *Rab11* (*ro-Rab5^N142I^* and *ro-Rab11^N124I^*). Rab11^N124I^ has been shown to act as a dominant negative late in eye development, where it blocks transport of rhodopsin to rhabdomeres and formation of multivesicular bodies in late endosomes [45]. Neither transgene had an effect on eye development, even when present in as many as four copies (data not shown). These results suggest that neither *Rab5* nor *Rab11* is required for this Notch signaling event, but there are other plausible explanations for the failure of these transgenes to interfere with Notch signaling. For example, expression levels that are too low for effective competition with wild-type proteins.

To overcome the problem in interpreting results obtained with dominant negative transgenes, we wanted to generate *Rab5-* or *Rab11-* (null) clones in the eye disc. *Rab5* null clones have an overgrowth phenotype that would obscure a Notch signaling defect [46]. *Rab11* null clones in the eye have not been reported, but we were able to generate them, and they were not hypertrophic (see below). The *Rab11* null allele we used, *Rab11^FRT^*, has a deletion of the promoter and first two exons, and produces no protein [47]. We used *Rab11* null clones to ask whether or not well-characterized signaling events in the eye disc required Rab11. The adult *Drosophila* eye develops from the larval eye imaginal disc, a monolayer epithelium [48]. Rows of ommatidia assemble stepwise posterior to the morphogenetic furrow, as it moves from the posterior to the anterior of the disc. The first cells to join the facets are the eight photoreceptors (R1-R8), and they do so in an invariant order in every ommatidium. Nearly every step of ommatidial assembly involves Notch signaling [49], [50], and so elimination of the Notch pathway in clones of mutant cells is catastrophic to eye development. In *Notch-* clones, no cells are specified as photoreceptors because Notch signaling is required anterior to the furrow to give cells neural potential, a process called proneural enhancement [51]. In *Delta-* clones, there are no photoreceptors in the middle of the clone. At the clone border, however, *Delta-* cells do become photoreceptors because they receive Notch signals from adjacent wild-type cells. Discrete ommatidia do not form within the clone because subsequent lateral inhibitory signaling cannot occur between adjacent *Delta-* cells, and the result is that too many cells adopt neural fate [51]. Clones of either *lqf-* (*liquid facets* [*lqf*] is the *Drosophila* epsin gene [44]) or *auxilin-* (*aux-*) cells in the eye disc appear identical to *Delta-* clones, consistent with the idea that epsin and auxilin are required in the signaling side of the Notch pathway [30], [33]. In accord with the developmental mutant phenotype, reporters for Notch activation are not expressed at all in *N-* cell clones, and are expressed in *Dl-, lqf-*, or *aux-* clones only in cells at the clone border, adjacent to wild-type cells that can signal [30,33,34,51, and see below].

**Figure 1:**
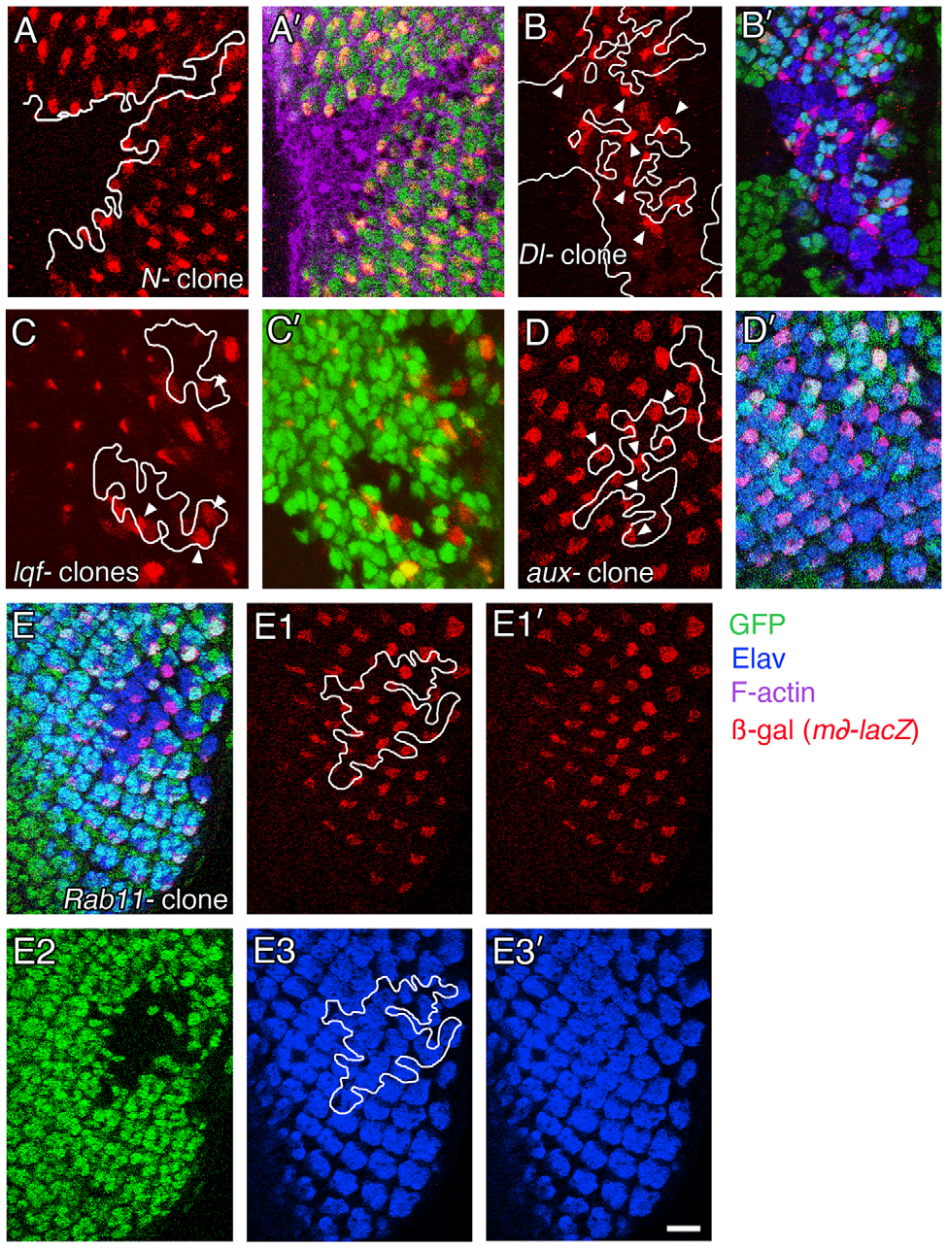
*Rab11* is not required for Notch signaling in eye discs. Confocal microscope images of third instar larval eye discs with clones of mutant cells are shown. The discs are immunolabeled to reveal Notch activation (anti-ßgal), photoreceptor cell nuclei (anti-Elav), and F-actin (phalloidin). Homozygous mutant cell clones are marked by the absence of nuclear GFP expression. Clones are outlined in white. Arrow heads point to some of the mutant cells within the clones that express ß-gal, indicating that Notch is activated. (A,A’) A *Notch* null (*N-*) clone was generated in larvae of the genotype *N^55e11^ FRT19A/ubi-ngfp FRT19A; ey-gal4, UAS-flp/+; m∂-lacZ/+.* (B,B’) A *Delta* null (*Dl-*) clone was generated in larvae of the genotype *ey-flp;m∂-lacZ/+; FRT82B Dl^rev10^/FRT82B ubi-ngfp* (C,C’) *lqf-* clones generated in larvae of the genotype *eyflp; m∂-lacZ/+; lqf^ARI^ FRT80B/ubi-ngfp FRT80B*. (D,D’) *aux-* clones were generated in larvae of the genotype ey-flp; m∂-lacZ/+; FRT^5-5Z3515^ aux^F956^*/FRT^5-5Z3515^ tub-ngfp. (E-E3’) The same *Rab11-* clone is shown in all panels, generated in larvae of the genotype *ey-flp; m∂-lacZ/+; Rab11^ΔFRT^/FRT5377 Hrb98DE::GFP*. Scale bar 20 µm.

We tested whether or not *Rab11-* (null) clones in eye discs would suffer severe defects in early ommatidial assembly, and whether or not *Rab11-* cells, especially those in the middle of the clone, would activate Notch. First, we observed *Rab11-* clones in eye discs immunostained with anti-Elav, which labels photoreceptor nuclei [52]. We found that compared with the calamitous effect on development in *N-, Dl-, lqf-,* or *aux-* clones [30], [33], [34], [51], ommatidial assembly was not obviously disrupted within the *Rab11-* clones; discrete ommatidia were present in the middle of the clone and at the clone borders (Fig. 1E, E2-E3’). This is consistent with results of similar experiments performed with *Rab11* hypomorphs, where eye morphology defects observed were due mainly to late events: cell death and the failure to form light-gathering rhabdomeres [45], [53]. These eye discs also contain a reporter transgene called *m∂-lacZ*, which is transcribed in R4 when Notch is activated in response to Delta signaling by R3 [54], [55]. This Notch signaling event distinguishes R3 and R4 [54]–[56]. No cells in *Notch-* (null) clones expressed *m∂-lacZ* (Fig 1A, A’), while *Delta-* (null) cells did express *m∂-lacZ*, but only when they were adjacent to wild-type cells at the clone edges (Fig. 1B, B’). We found that like *Delta-* cells, *lqf-* (null) or *aux-* (null) cells at the clone edge activated *m∂-lacZ* (Fig. 1C–D’). This result is consistent with other evidence that *lqf+* and *aux+* function in the signaling cells [27], [31], [33], [34], [40], and was important to show here because the marker used to assess Notch activation in *lqf-* or *aux-* eye clones previously [31], [33], [34], [40] was sometimes expressed in the absence of Notch activation [51]. In *Rab11-* cells, the pattern of *m∂-lacZ* expression was undisrupted; Notch was activated in the middle of the clone as well as at the edges (Fig. 1E–E2). The *m∂-lacZ* marker also revealed that the clusters inside *Rab11-* clones were at least normal enough that R4s were neatly spaced within the clone (Fig. 1E1, E1’). We conclude that *Rab11* is not required for several Notch signaling events in the eye disc – proneural enhancement, lateral inhibition, and R3/R4 signaling – all of which require epsin.

Although *Rab5* and *Rab11* are required for Notch signaling in *Drosophila* sensory organ precursor cells [15]–[17], it has been shown recently that female germline cells signal without either GTPase [40]. The observation here that *Rab11* is not required for several Notch signaling events in somatic cells indicates that the ability of a cell to signal independent of *Rab11* is not peculiar to the germline. Moreover, the eye disc is an epithelium, and thus the requirement for Rab11 in Notch signaling is not a general feature of epithelial cells. In addition, as the germline experiments were performed with a *Rab11* dominant negative transgene, residual *Rab11*+ activity could potentially have accounted for the results. Here, we remove all doubt that cells devoid of Rab11 may activate Notch in their neighbors.

### *auxilin* was not required for clathrin-independent Notch signaling in the ovary

Auxilin is known to be required for Notch signaling in the eye, wing, and embryo [31]–[34]. Strong genetic interactions between *clathrin heavy chain* (*chc*) and *lqf* [44], and the requirement for *aux* in signaling cells [31]–[34] suggested that epsin promotes clathrin-mediated endocytosis of ligand in signaling cells. Therefore, we were puzzled by the observation that for signaling by female germ-line cells, epsin is needed, but clathrin is dispensable [40]. One possibility suggested by this observation is that epsin likewise promotes clathrin-independent endocytosis of ligand in imaginal discs, and that in imaginal discs and embryos, auxilin and possibly also clathrin perform functions other than clathrin-mediated endocytosis. Alternatively, as epsin is known to facilitate both kinds of endocytic pathways [41]–[43], epsin may promote ligand endocytosis through a clathrin-independent pathway in female germline cells, and through a clathrin-dependent pathway in imaginal discs. In this scenario, auxilin would perform its known function in clathrin dynamics, which is uncoating clathrin-coated vesicles after internalization [39].

**Figure 2:**
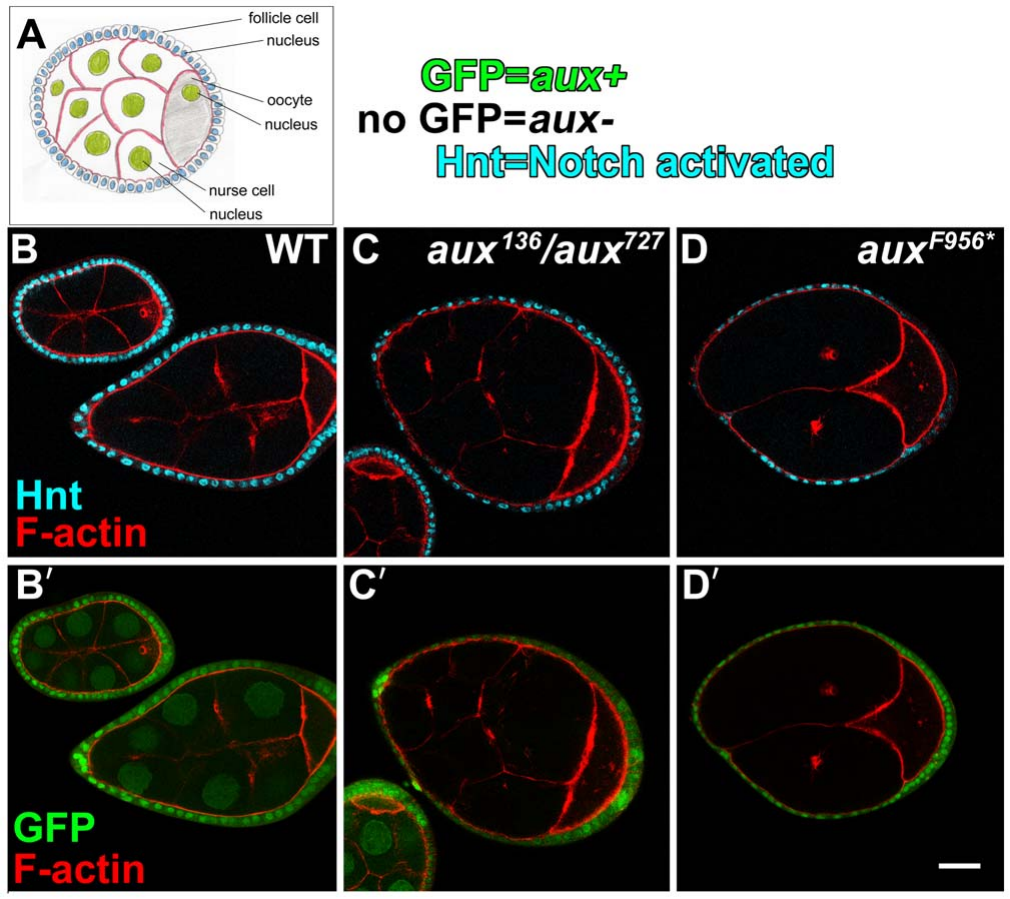
Female germline cells do not require *auxilin* to send Delta signals to follicle cells. (A) A diagram of an oocyte/nurse cell complex (stage 6–7) is shown. The fifteen nurse cells are diploid, and the cytoplasms of the nurse cells and the oocyte are interconnected by cytoplasmic bridges. (B–D’) Confocal microscope images of oocyte/nurse cell complexes are shown. The complexes were immunolabeled to reveal Notch activation in the follicle cells (anti-Hnt) and F-actin (phalloidin). Homozygous mutant cell nuclei are marked by the absence of GFP. (B,B’) Wild-type (WT) complexes are shown. Notch is activated in the follicle cells. (C,C’) A mosaic complex with *aux-* germ-line cells and *aux+* follicle cells is shown. Notch was activated in the follicle cells. The clone was generated in females of the genotype *hs-flp/+; ubi-gfp tub-aux FRT40A/FRT40A; aux^136^/aux^727^*. (D,D’) As in (C,C’), except the genotype was hs-flp/+; FRT^5-5Z3515^, aux^F956^*/FRT^5-5Z3515^, ubi-ngfp. Reduced levels of Hnt were seen at the poles of the *aux+/aux-* mosaic oocyte/nurse cell complexes, as was also observed in *Chc+/Chc-* mosaics [SLW and DB, unpublished observation]. This is quite distinct, however, from the absence of Hnt throughout the follicle epithelium observed with *lqf-* or *Dl-* germ line clones [40]. Scale bar 20 µm.

One way to distinguish between these two alternatives is to determine if the function of auxilin in Notch signaling is separable from the function of clathrin, and so we tested whether or not *aux+* was required in the female germline. In the ovary, the sixteen germline cells in the nurse cell/oocyte complex signal to surrounding somatic follicle cells at stage 6 of oogenesis, and Notch receptor activation may be monitored by expression of the target gene Hindsight (Hnt) (Fig. 2A) [40], [46]. In wild-type ovaries, Hnt is present in the nuclei of all surrounding follicle cells following stage 6 (Fig. 2B,B’) [40], [46]. In mosaic ovaries in which the follicle cells are *aux+* and the germline cells are *aux-* (null), the follicle cells nevertheless express Hnt (Fig. 2C–D’). Identical results were observed previously in ovaries mosaic for *Chc+* and *Chc-* cells [46] (see legend to Fig. 2). The same results were obtained using two different *aux-* backgrounds: *aux^136^/aux^727^* or aux^F956*^ homozygotes. *aux^136^* [32], [33] and aux^F956*^ [34] have nonsense mutations positioned between the codons for the clathrin binding domain and the J domains, which binds Hsc70. Thus, C-terminally truncated auxilin proteins that could in theory be produced would lack the J domain, which is essential for auxilin function in Notch signaling [33], [34]. *aux^727^* has a nonsense mutation early in the open reading frame, and an N-terminally truncated protein containing both the clathrin binding and J domains, produced by translation reinitiation, could function in Notch signaling [33], [34]. No auxilin protein from *aux^727^* was detectable with immunofluorescence using an auxilin antibody, and the genetic behavior of *aux^727^* was indistinguishable from that of *aux^136^* [34]. Thus, we conclude that the germline cells, which do not require clathrin for signaling, also do not require auxilin. This result indicates that germline and eye and wing disc cells simply internalize ligand through different endocytic pathways. Thus, the requirement for clathrin and auxilin in eye and wing discs most likely means that auxilin regulates clathrin dynamics in Notch signaling cells in the eye disc.

### Overexpression of *clathrin heavy chain* and *liquid facets* suppressed the semi-lethality and severe eye defects caused by strong *auxilin* mutations

The requirement for auxilin by the signaling cells provides a tool for testing the recycling model. Auxilin uncoats clathrin-coated vesicles, an expected prerequisite for fusing of newly endocytosed vesicles with the sorting endosome and subsequent transit through an endocytic pathway [39]. Auxilin activity, however, in addition to producing uncoated endocytic vesicles, also produces free clathrin. Indeed, free clathrin is depleted in the absence of auxilin [57], [58], and Delta endocytosis is inefficient in *aux* mutants [33]. Thus, it is possible that auxilin is required by signaling cells not to provide uncoated ligand-containing vesicles, but to provide free clathrin for use in the internalization step. If so, then providing free clathrin through different means should obviate the need for auxilin in signaling cells. Indeed, it was observed that *Chc+* overexpression partially suppressed the Notch signaling defects in eyes (and wings) associated with strong *aux* mutants [33]. Here, we tested the extent to which the lethality associated with *aux* mutations is also be suppressed by *Chc+* overexpression. In addition, we tested whether or not epsin overexpression also suppresses the *aux* mutant phenotype, and if the extent of suppression would be increased by by co-overexpressing clathrin heavy chain and epsin.

**Figure 3:**
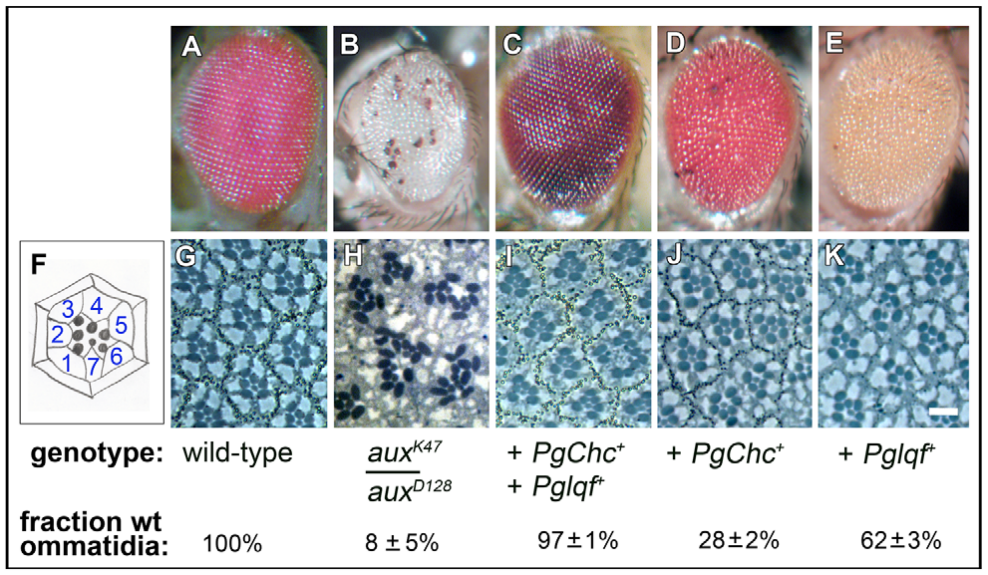
Overexpression of clathrin heavy chain and/or epsin suppresses the adult eye defects in *aux* loss-of-function mutants. (A–E) Light micrographs of adult external eyes of the genotypes indicated beneath are shown. (F) A diagram of an apical tangential section of a single ommatidium is shown. The numbers are photoreceptor cells R1 – R7. The black circular projections from each cells are the light-gathering organelles called rhabdomeres. The hexagonal shape is formed by pigment cells. (G–K) Small fields of apical tangential sections of adult eyes are shown. (H) Ommatidia of *aux* hypomorphs are usually disorganized, and often have extra photoreceptors. (I–K) Addition of genomic DNA transgenes that express *Chc+* or *lqf+* suppresses the eye morphology defects of *aux* hypomorphs. The fraction of phenotypically wild-type (wt) ommatidia was determined by observing 300–500 ommatidia in 4–5 eyes of each genotype. The error is one standard deviation. Scale bar 10 µm (G–K) and 60 µm (A–E).

First, we wondered how well the lethality of *aux* mutants, presumably caused by the failure of Notch signaling in early development [31], was suppressed by *Chc+* overexpression. Heterozygotes for one weak missense mutation and one strong nonsense mutation in *aux* (*aux^K47^*/*aux^D128^*) [32] rarely reach adulthood when grown at 25°C (Table 1). In addition, adult escapers have severely malformed imaginal disc-derived structures [32], including their eyes (Fig. 3A,B,F–H). Addition to the *aux^K47^*/*aux^D128^* flies of a transgene containing a genomic DNA copy of the *Chc+* gene (*PgChc+*) that can substitute for the endogenous *Chc+* gene [33] increases the eclosion frequency of adults markedly (Table 1). Also, as reported previously [33], the mutant eye phenotype of those rescued adults was suppressed somewhat (from 8% to 28% wild-type ommatidia) (Fig. 3D, J).

**Table 1:** Rescue of lethality of *aux* mutants by overexpression of epsin and/or clathrin heavy chain.

genotype^a^	# flies^c^	# expected^d^
*w; +/CyO; aux^K47^/aux^D128^*	2	0
*w; Pglqf+/+; aux^K47^/aux^D128^*	84	61
*w; PgChc+/CyO; aux^K47^/aux^D128^*	44	61
*w; PgChc+/Pglqf+; aux^K47^/aux^D128^*	52	61
*w; +/CyO; aux* b */TM6B*	69	122
*w; P glqf+/+; aux/TM6B*	114	122
*w; PgChc+/CyO; aux/TM6B*	193	122
*w; PgChc+/Pglqf+; aux/TM6B*	114	122
**total**	672	671

a The flies of the genotypic classes listed were obtained from crosses of three *w; gChc+/+; aux^K47^/TM6B* males with eight *w; glqf+/CyO; aux^D128^/TM6B* virgin females, kept at 25°C, and transferred to new food vials every 2–3 days for 5 days. Flies with *glqf+* only were differentiated from *gChc+/glqf+* flies by the latter having darker eye color.

b *aux* means either *aux^K47^* or *aux^D128^*

c The important comparison is between the first row and the three rows beneath. Addition of either or both *Pglqf+* or *PgChc+* transgenes increases drastically the viability of *aux^K47^/aux^D128^* adults. It is not clear why the effect of both transgenes is not greater than the effect of a single transgene. One possibility, suggested by the expected frequency of adults (see d below) is that each transgene rescues viability completely. In this case, the differences from expectation would be due to the effects of other aspects of the genotype, such as the presence or absence of *CyO*, and transgene insertion sites.

d The expected numbers were calculated making three simplifying assumptions: (1) *aux^K47^/aux^D128^* is completely lethal; (2) one copy of either transgene rescues viability fully; (3) no aspect of the genotype other than *aux^K47^/aux^D128^* affects viability.

Next, we wondered whether epsin overexpression, either alone or in combination with *Chc+* overexpression, would suppress the *aux^K47^*/*aux^D128^* mutant phenotype. We reasoned that if epsin links ligand to clathrin, it may be freed along with clathrin when auxilin uncoats clathrin from newly endocytosed vesicles. Alternatively, increased epsin levels in *aux* mutants may result in more efficient plasma membrane localization of the remaining free clathrin. We found that a transgene with a genomic DNA copy of the *lqf+* gene (*Pglqf+*) that complements *lqf* null mutants (similar to the transgene in ref. 44; X. X. and J.A.F., manuscript in preparation) rescued the lethality of *aux^K47^*/*aux^D128^* mutants (Table 1) and suppressed their mutant eye phenotype even better than *PgChc+* did (62% wild-type ommatidia) (Fig. 1E,K). Moreover, *aux^K47^*/*aux^D128^* flies carrying both *Pglqf+* and *PgChc+* had remarkably normal-appearing eyes (97% wild-type ommatidia) (Fig. 3C,I). However, no increase in viability was detected in these flies above the level observed with *Pglqf+* alone (Table 1; see also legend).

Thus, a single extra copy of either the *Chc+* gene or the *lqf+* gene suppressed the *aux* mutant phenotype, including lethality, significantly. Remarkably, a single extra copy of both the *Chc+* and *lqf+* genes suppressed nearly completely the severe morphological abnormalities due to Notch signaling defects in *aux* mutants. This indicates that supplying free clathrin heavy chain and additional epsin to the cells bypasses the large part of the need for auxilin in Notch signaling. We conclude that the primary role of auxilin in Notch signaling cells is to maintain the pool of free clathrin, and possibly also epsin.

## Discussion

There are three major results of this work. First, we found that *Rab11* is not required for several Notch signaling events in the developing *Drosophila* eye that require epsin and auxilin. Thus, as in the female germline cells, ligand recycling, at least via a *Rab11*-dependent pathway, is not necessary for Notch signaling in the eye disc. Second, we found that the one Notch signaling event presently known to be clathrin-independent is also auxilin-independent. This result reinforces the idea that rather than performing some obscure function, the role of auxilin in Notch signaling cells is to regulate clathrin dynamics. Finally, we showed that overexpression of both clathrin heavy chain and epsin rescues to nearly normal the severely malformed eyes and semi-lethality of *aux* hypomorphs. Presumably, vesicles uncoated of clathrin fuse with the sorting endosome, and so it seems reasonable to assume that uncoating clathrin-coated vesicles containing ligand is preprequisite for trafficking ligand through endosomal pathways. Thus, if ligand endocytosis is prerequisite to recycling, efficient production of uncoated vesicles would be required. In *aux* mutants with severe *Notch*-like mutant phenotypes, clathrin vesicle uncoating is inefficient. We presume that this remains so even when clathrin and epsin are overexpressed, yet the eye defects and lethality are nearly absent. Thus, we reason that auxilin is required not for efficient production of uncoated vesicles *per se*, but for the other product of auxilin activity – free clathrin (and possibly also free epsin). Taken together, these results argue strongly that at least in some cell types, the fundamental role of Notch ligand endocytosis is not ligand recycling.

Is it possible that the fundamental mechanism of Notch signaling is so completely distinct in different cell types, that ligand endocytosis serves *only* to activate ligand via recycling in some cellular contexts, and *only* for exerting mechanical force on the Notch receptor in others? While formally possible, this is not parsimonious. Thus, we favor a model where the fundamental role of ligand endocytosis is to exert mechanical force on the Notch receptor. In addition, some cell types will also require ligand recycling. As no altered, activated form of ligand has yet been identified, while ligand transcytosis has been well-documented [15]–[17], [28], the most likely role of recycling is to relocalize ligand on the plasma membrane prior to Notch receptor binding.

## Materials and Methods

### *Drosophila* mutants and transgenes

The alleles and transgenes used are listed below. FlyBase id numbers (http://flybase.org/) are provided when available. Chromosomes and genotypes used in particular experiments are indicated in Figure Legends. Mutant alleles: aux^F956^* (FBal0240439), *aux^K47^* (FBal0197315), *aux^D128^* (FBal0197310), *aux^136^* (FBal0197311), *aux^727^*(FBal0197308), *Rab11^ΔFRT^* [47], *Dl^revF10^* (FBal0029366), *N^55e11^* (FBal0012701). Transgenes: *PgChc+* [33], *tub-aux* [33], *ey-flp* (FBti0015982), *m∂-lacZ* (on 2 and 3; FBtp0010977), *hs-flp^122^* (on X), *ubi-ngfp* (on X,2L,3R), *Hrb98DE::GFP* [47], *FRT82B* (FBti0002074), *FRT18A* (FBti0002070), *FRT40A* (FBti0002071), *FRT5377* [47], *FRT^5-5Z3515^* [34], *ey-gal4* (on 2), *UAS-flp* (on 2), *UAS-Rab11^N124I^* (FBal0190955). Transgenes generated in this work: *Pglqf+* (on 2), *ro-Rab5^N142I^* (multiple lines), *ro-Rab11^N124I^* (multiple lines).

### Transgene construction

#### *Pglqf+*

This construct is an ∼16,240 bp *Not I* – *Xho I* fragment of *Drosophila* genomic DNA containing the *lqf+* gene obtained from a subclone called 19G [44], with the C-terminal codons fused to Ala6-GFP, ligated into *pCaSpeR4* restricted with *Not I* and *Xho I*. The GFP tag was inserted using a two-step PCR method (X.X. and J.A.F., manuscript in preparation).

#### *ro-Rab5^N142I^*

Total RNA from 5 *w^1118^* females was isolated using TRI reagent (Molecular Research Center), and 5 µg was used for reverse transcription with SuperScriptII (Invitrogen). The primers used were Rab5F (5′-AAAGGCGCGCCATGGCAACCACTCCACGC-3′) and Rab5R (5′-AAAGGCGCGCCTCACTTGCAGCAGTTGTTCG-3′). The cDNA was diluted to 200 µl, and 2 µl was used as the template for the following PCR reactions. The mutant *Rab5* cDNA was generated in two steps. First, two PCR reactions were performed with mutagenic primers, Rab5CF (5′-GGCCGGCA**T**CAAGGCAG-3′) and Rab5NR (5′-CTGCCTTG**A**TGCCGGCC-3′). One reaction used the primer pairs F and NR, and the other used R and CF. Next, the amplification products from each reaction were mixed, and used together as a template for PCR with primers F and R. The resulting amplification product was ligated as an *Asc I* fragment into *BluescriptIIKS+* (Stratagene) with its *Bam HI* site changed to *Asc I,* an its DNA sequence was verified. Finally, an ∼660 bp *Asc I* fragment containing the *Rab5^N142I^* cDNA was ligated into *pRO* [59].

#### *ro-Rab11^N124I^*

The mutant *Rab11* cDNA was obtained by PCR using as template genomic DNA from flies containing *UAS-Rab11^N124I^* [45], and the primers Rab11F (5′-AAAGGCGCGCCATGGGTGCAAGAGAAGACGA-3′) and Rab11R (5′-AAAGGCGCGCCTCACTGACAGCACTGTTTGC-3′). The resulting ∼660 bp amplification product was ligated as an *Asc I* fragment into *pUAS_t_ –XA* [57] restricted with *Asc I.*

### Analysis of eyes

Plastic sectioning of adult eyes was performed as described [60], and sections were viewed and photographed with a Zeiss Axioplan equipped with an Axiocam HRc. Eyes were photographed in whole flies using an Olympus SZX12 microscope equipped with a SPOT idea (Diagnostic Instruments) camera. For immunostaining, eye discs were fixed in PEMS and antibody incubations and washes were in PBST as described [61]. Primary antibodies were from the Developmental Studies Hybridoma Bank (DSHB): rat monoclonal anti-Elav (1∶1), and mouse monoclonal anti-ßgal (1∶50). Secondary antibodies were: 568-AlexaFluor goat anti-mouse (1∶200) (Invitrogen), and Cy5-AffiniPure goat anti-rat (1∶200) (Jackson ImmunoResearch). 633-AlexaFlour phalloidin (Invitrogen) was also used (15 µl of a 300 U/1.5 ml methanol stock solution). Immunofluorescent eye discs were photographed with a Leica TCSSP2 or SP2AOBS confocal microscope. Images were processed with Adobe Photoshop.

### Analysis of germline clones

*aux-* germ line clones were generated by heat shocking first to third instar larvae at 37°C for 2 hours on 2 consecutive days. Adult females of the appropriate genotype were collected upon eclosion. The females were fed on yeast in the presence of males for 2 days, flipped onto fresh yeast for 2 more days, and then their ovaries dissected. Egg chambers were fixed in 4% formaldehyde in 1X phosphate-buffered saline (PBS) for 15 minutes and washed with PBS. Primary antibody staining was performed in 1X PBS + 0.3% Triton-100 (PBT3) containing 5% normal goat serum overnight at 4°C, followed by washing with PBT, staining with secondary antibodies, and mounting in antifade reagent (Invitrogen). The following antibodies were used: mouse anti-Hindsight at a dilution of 1∶50 (DSHB) and 647-AlexaFluor donkey anti-mouse (Invitrogen). Cells were also labeled with TRITC-phalloidin (Sigma) at 1∶200 to detect F-actin. Images were collected using a Leica TCS confocal microscope and assembled using Adobe Photoshop. Single sections are shown for each sample.

## References

[1] Scita G, Di Fiore PP (2010). The endocytic matrix.. Nature.

[2] Le Borgne R, Bardin A, Schweisguth F (2005). The roles of receptor and ligand endocytosis in regulating Notch signaling.. Development.

[3] Le Borgne R (2006). Regulation of Notch signaling by endocytosis and endosomal sorting.. Curr Opin Cell Biol.

[4] Chitnis A (2006). Why is Delta endocytosis required for effective activation of Notch?. Dev Dyn.

[5] Nichols JT, Miyamoto A, Weinmaster G (2007). Notch signaling – constantly on the move.. Traffic.

[6] Fortini ME (2009). Notch signaling: the core pathway and its posttranslational regulation.. Dev Cell.

[7] Furthauer M, Gonzalez-Gaitan M (2009). Endocytic regulation of Notch signaling during development.. Traffic.

[8] Bray SJ (2006). Notch signaling: a simple pathway becomes complex.. Nat Rev Mol Cell Biol.

[9] Sun X, Artavanis-Tsakonas S (1997). Secreted forms of Delta and Serrate define antagonists of Notch signaling in *Drosophila.*. Development.

[10] Parks AL, Klueg KM, Stout JR, Muskavitch MAT (1995). Ligand endocytosis drives receptor dissociation and activation in the Notch pathway.. Development.

[11] Nichols JT, Miyamoto A, Olsen SL, D'Souza B, Yao C (2007). DSL ligand endocytosis physically dissociates Notch1 heterodimers before activating proteolysis can occur.. J Cell Biol.

[12] Gordon WR, Vardar-Ulu D, Histen G, Sanchex-Irizarry C, Aster JC (2007). Structural basis for autoinhibition of Notch.. Nat Struct Mol Biol.

[13] Matsuda M, Chitnis AB (2009). Interaction with Notch determines endocytosis of specific Delta ligands in zebrafish neural tissue.. Development.

[14] Stenmark H (2009). Rab GTPases as coordinators of vesicle traffic.. Nat Rev Mol Cell Biol.

[15] Jafar-Nejad J, Andrews HK, Acar M, Bayat B, Wirtz-Peitz F (2005). Sec15, a component of the exocyst, promotes Notch signaling during the saymmetric division of *Drosophila* sensory organ precursors.. Dev Cell.

[16] Emery G, Hutterer A, Berdnik D, Mayer B, Wirtz-Peitz F (2005). Asymmetric Rab11 endosomes regulate Delta recycling and specify cell fate in the *Drosophila* nervous system.. Cell.

[17] Rajan A, Tien A-C, Haueter CM, Schulze KL, Bellen HJ (2009). The Arp2/3 complex and WASp are required for apical trafficking of Delta into microvilli during cell fate specification of sensory organ precursors.. Nat Cell Biol.

[18] Lai EC, Deblandre GA, Kintner C, Rubin GM (2001). *Drosophila* Neuralized is a ubiquitin ligase that promotes the internalization and degradation of Delta.. Dev Cell.

[19] Deblandre GA, Lai EC, Kintner C (2001). Xenopus Neuralized is a ubiquitin ligase that interacts with XDelta1 and regulates Notch signaling.. Dev Cell.

[20] Pavlopoulos E, Pitsouli C, Klueg KM, Muskavitch MAT, Moschonas NK (2001). Neuralized encodes a peripheral membrane protein involved in Delta signaling and endocytosis.. Dev Cell.

[21] Yeh E, Dermer M, Commisso C, Zhou L, McGlade CJ (2001). Neuralized functions as an E3 ubiquitin ligase during *Drosophila* development.. Curr Biol.

[22] Itoh M, Kim CH, Palardy G, Oda T, Jiang YJ (2003). Mind bomb is a ubiquitin ligase that is essential for efficient activation of Notch signaling by Delta.. Dev Cell.

[23] Pitsouli C, Delidakis C (2005). The interplay between DSL proteins and ubiquitin ligases in Notch signaling.. Development.

[24] Lai EC, Roegiers F, Qin X, Jan YN, Rubin GM (2005). The ubiquitin ligase *Drosophila* Mindbomb promotes Notch signaling by regulating the localization and activity of Serrate and Delta.. Development.

[25] Le Borgne R, Remaud S, Hamel S, Schweisguth F (2005). Two distinct E3 ubiquitin ligases have complementary function in the regulation of Delta and Serrate signaling in *Drosophila*.. PLoS Biol.

[26] Wang W, Struhl G (2005). Distinct roles for Mind bomb, Neuralized, and epsin in mediating DSL endocytosis and signaling in *Drosophila.*. Development.

[27] Wang W, Struhl G (2004). *Drosophila* epsin mediates a select endocytic pathway the DSL ligands must enter to activate Notch.. Development.

[28] Benhra N, Vignaux F, Dussert A, Schweisguth F, Le Borgne R (2010). Neuralized promotes basal to apical transcytosis of Delta in epithelial cells.. Mol Biol Cell.

[29] Heuss SF, Ndiaye-Lobry D, Six EM, Israel A, Logeat F (2008). The intracellular region of Notch ligands Dll1 and Dll3 regulates their trafficking and signaling activity.. J Cell Sci.

[30] Overstreet E, Fitch E, Fischer JA (2004). Fat facets and Liquid facets promote Delta endocytosis and Delta signaling in the signaling cells.. Development.

[31] Hagedorn EJ, Bayraktar JL, Kandachar VR, Bai T, Englert DM (2006). *Drosophila melanogaster* auxilin regulates the internalization of Delta to control activity of the Notch signaling pathway.. J Cell Bio.

[32] Eun SH, Lea K, Overstreet E, Stevens S, Lee J-H (2007). Identification of genes that interact with *Drosophila liquid facets*.. Genetics.

[33] Eun SH, Banks SML, Fischer JA (2008). Auxilin is essential for Delta signaling.. Development.

[34] Kandachar V, Bai T, Chang HC (2008). The clathrin-binding motif and the J-domain of *Drosophila* auxilin are essential for facilitating Notch ligand endocytosis.. BMC Dev.

[35] Bai T, Seebald JL, Kim KE, Ding HM, Szeto DP (2010). Disruption of zebrafish cyclin G-associated kinase (GAK) function impairs the expression of Notch-dependent genes during neurogenesis and causes defects in neuronal development.. BMC Dev Biol.

[36] Tian X, Hansen D, Schedl T, Skeath JB (2004). Epsin potentiates Notch pathway activity in *Drosophila* and *C. elegans*.. Development.

[37] Chen H, Ko G, Zatti A, Di Giacomo G, Liu L (2009). Embryonic arrest at midgestation and disruption of Notch signaling produced by the absence of both epsin 1 and epsin 2 in mice.. Proc Natl Acad Sci USA.

[38] Horvath CA, Vanden Broeck D, Boulet GA, Bogers J, De Wolf MJ (2007). Epsin: inducing membrane curvature.. Int J Biochem Cell Biol.

[39] Eisenberg E, Greene LE (2007). Multiple roles of auxilin and Hsc70 in clathrin-mediated endocytosis.. Traffic.

[40] Windler SL, Bilder D (2010). Endocytic internalization routes required for Delta/Notch signaling.. Curr Biol.

[41] Chen H, Fre S, Slepnev VI, Capua MR, Takei K (1998). Epsin is an EH-domain-binding protein implicated in clathrin-mediated endocytosis.. Nature.

[42] Sigismund S, Woelk T, Puri C, Maspero E, Tacchetti C (2005). Clathrin-independent endocytosis of ubiquitinated cargos.. Proc Natl Acad Sci USA.

[43] Chen H, De Camilli P (2005). The association of epsin with ubiquitinated cargo along the endocytic pathway is negatively regulated by its interaction with clathrin.. Proc Natl Acad Sci USA.

[44] Cadavid ALM, Ginzel A, Fischer JA (2000). The function of the *Drosophila* Fat facets deubiquitinating enzyme in limiting photoreceptor cell number is intimately associated with endocytosis.. Development.

[45] Satoh AK, O'Tousa JE, Ozaki K, Ready DF (2005). Rab11 mediates post-Golgi trafficking of rhodopsin to the photosensitive apical membrane of *Drosophila* photoreceptors.. Development.

[46] Vaccari T, Lu H, Kanwar R, Fortini ME, Bilder D (2008). Endosomal entry regulates Notch receptor activation in *Drosophila melanogaster* J Cell Biol.

[47] Bogard N, Lan L, Xu J, Cohen RS (2007). Rab11 maintains connections between germline stem cells and niche cells in the *Drosophila* ovary.. Development.

[48] Wolff T, Ready DF, Bate M, Martinez Arias A (1993). Pattern formation in the *Drosophila* retina.. The development of *Drosophila melanogaster*.

[49] Roignant J-Y, Treisman JE (2009). Pattern formation in the *Drosophila* eye disc.. Int J Dev Biol.

[50] Carthew RW (2007). Pattern formation in the *Drosophila* eye.. Curr Opin Genet Dev.

[51] Baker NE, Yu S-Y (1996). Proneural function of neurogenic genes in the developing *Drosophila* eye.. Curr Biol.

[52] Robinow S, White K (1991). Characterization and spatial distribution of the ELAV protein during *Drosophila melanogaster* development.. J Neurobiol.

[53] Alone DP, Tiwari AK, Mandal L, Li M, Mechler BM (2005). *Rab11* is required during *Drosophila* eye development. Int. J Dev Bio.

[54] Fanto M, Mlodzik M (1999). Asymmetric activation specifies photoreceptors R3 and R4 and planar polarity in the *Drosophila* eye.. Nature.

[55] Cooper MTD, Bray SJ (1999). Frizzled regulation of Notch signaling polarizes cell fate in the *Drosophila* eye.. Nature.

[56] Tomlinson A, Struhl G (1999). Decoding vectorial information from a gradient: sequential roles of the receptors Frizzled and Notch in establishing planar polarity in the *Drosophila* eye.. Development.

[57] Gall WE, Higginbotham MA, Chen C-Y, Ingram MF, Cyr DM (2000). The auxilin-like phosphoprotein Swa2p is required for clathrin function in yeast.. Curr Biol.

[58] Pishavee B, Costaguta G, Yeung BG, Ryazantsev S, Greener T (2000). A yeast DNA J protein required for uncoating of clathrin coated vesicles in vivo.. Nat Cell Biol.

[59] Huang Y, Fischer-Vize JA (1996). Undifferentiated cells in the developing Drosophila eye influence facet assembly and require the Fat facets deubiquitinating enzyme.. Development.

[60] Tomlinson A, Ready DF (1987). Cell fate in the *Drosophila* ommatidium.. Dev Biol.

[61] Fischer-Vize JA, Rubin GM, Lehmann R (1992). The *fat facets* gene is required for *Drosophila* eye and embryo development.. Development.

